# Efficacy and safety of tailin formulation combined with continuous low-dose antibiotic therapy in patients with recurrent urinary tract infection: A multicenter, randomized, controlled clinical trial

**DOI:** 10.3389/fphar.2022.968980

**Published:** 2022-09-14

**Authors:** Tonglu Li, Yingru Xu, Gang Yuan, Wen Lu, Guihua Jian, Xuezhong Gong

**Affiliations:** ^1^ Department of Nephrology, Shanghai Municipal Hospital of Traditional Chinese Medicine, Shanghai University of Traditional Chinese Medicine, Shanghai, China; ^2^ Department of Nephrology, Shanghai Baoshan District Hospital of Integrated Traditional Chinese and Western Medicine, Shanghai University of Traditional Chinese Medicine, Shanghai, China; ^3^ Department of Nephrology, Shanghai Sixth People’s Hospital, Shanghai Jiaotong University, Shanghai, China

**Keywords:** recurrent urinary tract infection, renal fibrosis, tailin formulation, continuous low-dose antibiotic therapy, traditional Chinese medicine, randomized controlled trial

## Abstract

Persistent inflammation associated with recurrent urinary tract infection (rUTI) is a crucial inducement of inflammation-driven renal fibrosis (IDRF). Although continuous low-dose antibiotic therapy (CLAT) is the common treatment for rUTI, its clinical efficacy remains unsatisfactory. Tailin formulation (TLF), a Chinese herbal formulation prescribed for treating rUTI, is effective in alleviating symptoms and reducing recurrence. This study was to evaluate the efficacy and safety of TLF combined with CLAT compared with CLAT used alone in patients with rUTI. In this multicenter, randomized, controlled clinical trial, patients were assigned (1:1) to receive either TLF + CLAT or CLAT for 12 weeks. The primary outcome was the effective rate at week 12 of the treatment. The secondary outcomes were the recurrent rate at week 4 and week 12 post treatment; the post-treatment changes in renal tubular injury markers (urinary N-acetyl-β-d-glucosaminidase (NAG) and β2-microglobulin (β2-MG)), profibrotic factors (urinary monocyte chemoattractant protein-1 (MCP-1) and transforming growth factor beta1 (TGF-β1)), and traditional Chinese medicine (TCM) symptoms, and vital signs indicators and serious adverse events (SAEs) were also monitored throughout the trial. A total of 195 patients were included in the final analysis. The TLF + CLAT group had a higher effective rate and a lower recurrence rate than the CLAT group (*p* < 0.01). Significant decrease of urinary NAG and β2-MG was observed in the TLF + CLAT group vs. CLAT group (*p* < 0.01), and similar changes were observed in profibrotic factors (urinary MCP-1 and TGF-β1) (*p* < 0.05), which indicated that TLF might have potential renal tubular protection and anti-fibrosis effects. Additionally, a positive correlation within a certain range was shown in the correlation analysis of medical history (months) of rUTI patients with urinary MCP-1 (r = 0.50, *p* < 0.05) and TGF-β1 (r = 0.78, *p* < 0.01). A significant difference was also observed in TCM symptoms (*p* < 0.01). There were no obvious adverse reactions that occurred during this study. We conclude that TLF combined with CLAT was superior to CLAT used alone in reducing rUTI recurrence, alleviating the non-infection-related physical symptoms and protecting renal tubular and anti-fibrosis, which suggests this novel therapy might be an available treatment with great promise in treating rUTI.

## 1 Introduction

rUTI is a common clinical refractory disease affecting millions of people across the globe annually (Geerlings, 2016), exerting considerable impact on patient's quality of life (Wagenlehner et al., 2018).

Inflammation of the urinary tract is the critical pathological mechanism of rUTI (Kaur and Kaur, 2021), which might result in renal tubular damage (Lorenzo-Gomez et al., 2020) and renal fibrosis (Lv et al., 2018) in those patients. However, there is insufficient evidence to reflect renal damage and inflammation-driven renal fibrosis (IDRF) as subsequent issues due to the rUTI. Urinary NAG and β2-MG are sensitive markers of tubular injury to predict acute kidney injury (AKI) (Ix and Shlipak, 2021). Elevated urinary NAG and β2-MG values have been described in patients suffering risk factors, including nephrotoxic drugs (Raduly et al., 2021). Therefore, presumably, renal damage attributable to rUTI could be evidenced by the increase of urinary NAG and β2-MG.

Persistent inflammation associated with rUTI is a crucial inducement of IDRF (Humphreys, 2018; Lv et al., 2018), involving complex interactions among multiple inflammatory cells and profibrotic cytokine signaling pathways. Critical profibrotic factors include MCP-1 and TGF-β1. Inflammation might promote fibrosis by inflammatory cells, which is tightly regulated by TGF-β1 (Humphreys, 2018). MCP-1 also plays an important role in the process of inflammation for it could attract other inflammatory factors/cells and has been found to be associated with renal fibrosis (Singh et al., 2021). Given that, changes of critical profibrotic factors (urinary MCP-1 and TGF-β1) in rUTI patients should be paid more attention.

In addition to the high prevalence and economic burden of rUTI, the presumable renal damage and IDRF attributed to rUTI in those patients bring an even greater challenge in the treatment of rUTI.

Antibiotic treatment including CLAT is the most common treatment for rUTI globally (Anger et al., 2019). Although CLAT is effective in inhibiting uropathogenic bacteria and shortening the duration of the infection-related symptoms in episodes, the clinical efficacy and safety of CLAT still remain unsatisfactory due to the presumable side effects and lack of long-term efficacy (Flower et al., 2016; Forbes et al., 2018). Consequently, alternative treatments are being considered, such as Chinese herbal medicine (CHM).

Based on 5,000 years of practice and experience, Chinese medicine is an essential part of healthcare in China. Comparable beneficial outcomes have also been reported for CHM products administered to patients with rUTI (Flower et al., 2019).

TLF (Gong et al., 2006) is a herbal prescription developed by Professor Gong Xuezhong for the treatment of rUTI, consisting of Radix Pseudostellariae (Taizishen), Radix Rehmanniae Recen (Shengdihuang), Sargentodoxa Cuneata (Daxueteng), and Polygonum Cuspidatum (Huzhang). Previous studies by our group have shown the possible roles for TLF in the treatment of rUTI (Gong et al., 2006; Gong et al., 2007; Gong et al., 2010). In animal experiments, we have built rat models of cystitis, acute pyelonephritis (APN), and chronic pyelonephritis (CPN) by *E. coli O*_*111*_ *B*_*4*_ successfully (Gong et al., 2003; Gong et al., 2004) and found that TLF might effectively protect renal tubules from injury, inhibit renal tubular and interstitial inflammation, and cures renal fibrosis in rats with UTI (Gong et al., 2006; Gong et al., 2010). In the clinical trial (Gong et al., 2007), we have found that compared with the levofloxacin group, urine NAG/Cr and β2-MG decreased significantly in the TLF group, suggesting that TLF could protect the renal tubular function of CPN patients.

Since the clinical efficacy of CLAT in the rUTI population is unsatisfactory, this study attempted to determine if there was a more effective alternative in patients with rUTI using LTF combined with CLAT. Moreover, given that persistent inflammation associated with rUTI is a crucial inducement of IDRF, we also explored the possible relationship between the critical profibrotic factors (TGF-β1, MCP-1) and the medical history (months) of rUTI patients in the present study to clarify if renal fibrosis can be associated with rUTI.

Here, we report the results of this study, hoping to provide reliable evidence-based medical evidence for treatment of rUTI and ease the burden for patients with rUTI.

## 2 Materials and methods

### 2.1 Study design

This trial ran from January 2021 to March 2022 and involved three tertiary hospitals (Shanghai Municipal Hospital of Traditional Chinese Medicine, Shanghai University of Traditional Chinese Medicine; Shanghai Sixth People’s Hospital, Shanghai Jiaotong University; Shanghai Baoshan District Hospital of Integrated Traditional Chinese and Western Medicine, Shanghai University of Traditional Chinese Medicine). Patients eligible for the trial in each trial site were assigned a randomized 1:1 ratio to receive LTF + CLAT or CLAT for 12 weeks. This study protocol was approved by the Institutional Ethics Committee (record number: 2020SHL-KY-47) and registered at the Chinese Clinical Trial Registry (registration number: ChiCTR-TRC-10001518).

### 2.2 Sample size

The sample size was calculated by PASS (version 15.0). Based on the previous trial (Li, 2018), 91 patients per group were needed to achieve 90% power to detect a difference between the group proportions of 0.18. Assuming an attrition rate of 10%, a planned recruitment target of 100 patients per group will be set.

### 2.3 Participants

According to the inclusion and exclusion criteria, rUTI patients diagnosed with the qi-yin of spleen and kidney deficiency and damp-heat adhesion syndrome by TCM will be considered potential participants.

### 2.4 Diagnostic criteria

#### 2.4.1 For UTI

Patients would be diagnosed with UTI if

1) urine culture: ≥10^5^ cfu/ml bacteria;
2) urinary sediment: white blood cell (WBC) > 10/HP, or with clinical symptoms of UTI.

#### 2.4.2 For rUTI

rUTI is defined as more than two UTIs in the last 6 months or more than three UTIs in the last 12 months, and the course of the disease is more than 2 years.

### 2.5 Inclusion/exclusion criteria

Eligibility criteria for inclusion were patients who were aged 18–70 years; history of rUTI (at least two episodes in the last 6 months or more than three episodes in the last 12 months); diagnosed with the qi-yin of spleen and kidney deficiency and damp-heat adhesion syndrome by TCM; no known allergies to the drugs to be prescribed; agreed to take part in the trial and sign informed consent.

Exclusion criteria were pregnancy or lactation; with an in-dwelling catheter; urethral syndrome; chronic kidney disease (CKD) stages IV–V (eGFR<30 ml/min); combined with serious heart and liver function damage or diabetes and other diseases which need immediate treatment; severe central nervous system disease; participating in other drug clinical trials or have participated in other clinical trials in the last 3 months.

### 2.6 Randomization and blinding

Patients eligible in each trial site were assigned randomly, using a computer-generated random number sequence. The division of the groups in this study will be blinded to all participants, investigators, and statisticians. The placebo and TLF are the same in the appearance of the drug package.

### 2.7 Intervention

Participants randomly assigned to the treatment group were administered TLF + CLAT for 12 weeks, and the participants assigned to the control group received CLAT + placebo for 12 weeks. Dosage and duration are shown in Table 1.

**TABLE 1:** Dosage and duration of drugs for the two group.

Treatment	Time					
	Week 1 and 2	Week 3 and 4	Week 5 and 6	Week 7 and 8	Week 9 and 10	Week 11 and 12
**TLF**	◇	◇	◇	◇	◇	◇
**TLF Placebo**	◆	◆	◆	◆	◆	◆
**CLAT**						
**Levofloxacin (0.1g, QN, po)**	◇◆			◇◆		
**Nitrofurantoin (0.1g, QN, po)**		◇◆			◇◆	
**Cefdinir (0.1g, QN, po)**			◇◆			◇◆

◇represents the administration of the TLF + CLAT group, ◆represents the administration of the CLAT group.

### 2.8 Outcomes

The primary outcome was the effective rate at week 12 of treatment. The second outcomes were the recurrence rate at weeks 4 and 12 after treatment; post-treatment changes in urinary NAG/Cr, β2-MG, TGF-β1, and MCP-1; furthermore, traditional Chinese medicine (TCM) symptoms were also scored; meanwhile, vital sign indicators and serious adverse events (SAEs) were monitored throughout the trial.

### 2.9 Follow-up

After treatment was completed, all patients had a follow-up of 12 weeks. Efficacy-related examinations and safety indicators were performed and collected during the treatment and follow-up period. Routine urine examination was conducted at week 0 of the treatment period and every 2 weeks during the treatment period and the follow-up period; cleaning middle urine cultivation was conducted at weeks 0 and 4 and 8 and 12 of the treatment period and weeks 4 and 12 post treatment. Urinary NAG/Cr, β2-MG, TGF-β1, and MCP-1 were measured at weeks 0 and 12 of the treatment period.

### 2.10 Statistical analysis

Original data were collected and verified by monitors at every visit. Continuous variables of the endpoints were compared with those at the baseline using an analysis of variance or Student’s t-test. After processing the categorical variables, the researchers used Fisher’s exact test, the chi-square test, or the Cochran–Mantel–Haenszel test to analyze the results. The continuous variables conforming to normality will be analyzed using Pearson’s correlation test, and those that do not conform to normality will be analyzed using the Spearman correlation test.

## 3 Results

**FIGURE 1:**
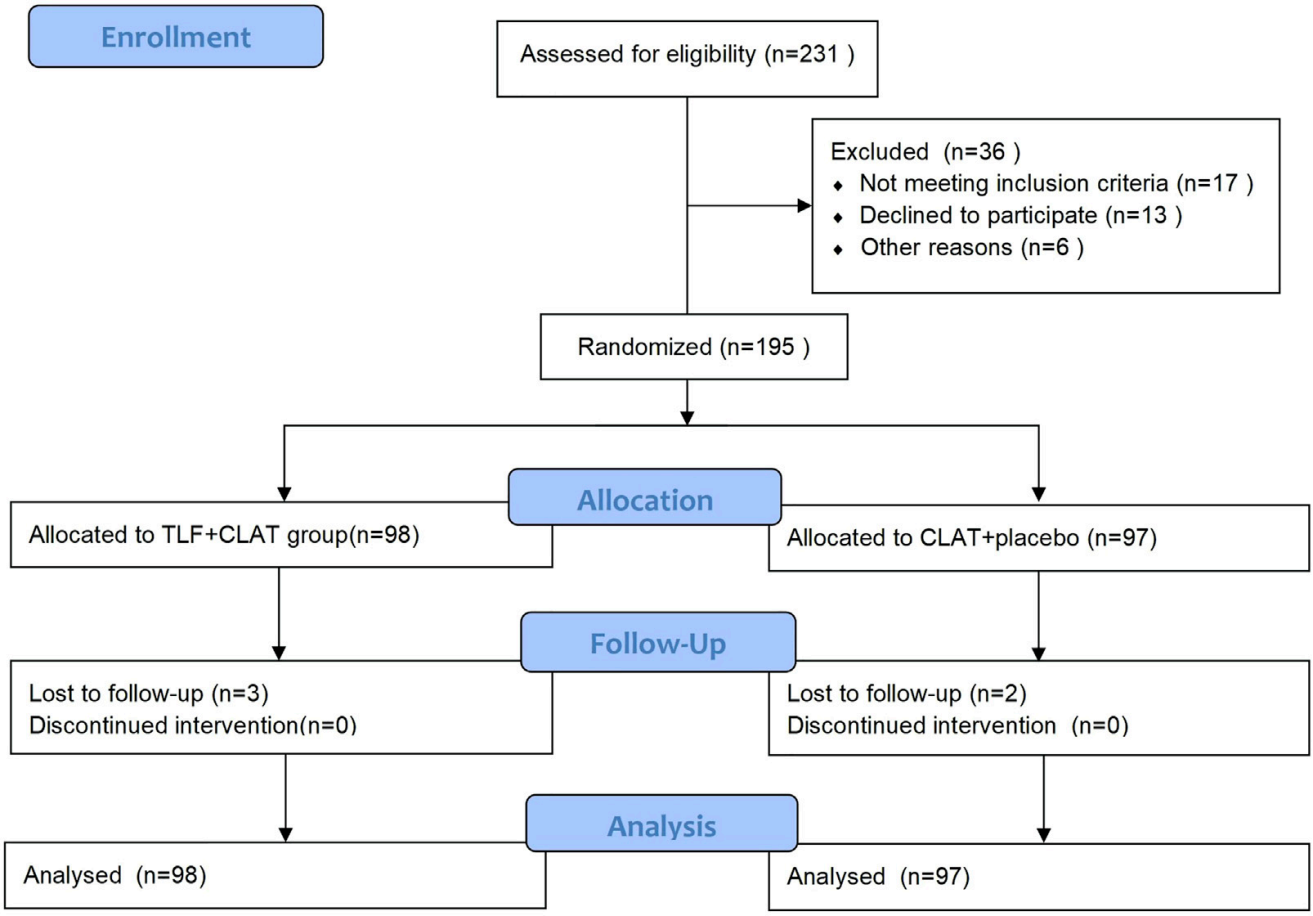
Participant flowchart.

During the recruitment period, patients with uncomplicated rUTI were randomized with a 1:1 ratio into two groups to receive TLF + CLAT or CLAT. Figure 1 shows a CONSORT flow chart. The final data analysis included 98 patients in the TLF + CLAT group and 97 in the CLAT group.

### 3.1 Baseline characteristics of patients

The patients were comparable between the three groups in terms of demographic and disease characteristics.

Table 2 shows that the mean age was 52.24 years (SD 13.92) in the TLF group and 53.01 years (SD 13.90) in the CLAT group; the percentage of females present was 69.39% (68/98) in the TLF group and 68.04% (66/97) in CLAT group; the mean medical history (recorded from the first diagnosis of rUTI) of rUTI patients was 43.85 months (SD 14.24) in the TLF group and 43.06 months (SD 12.76) in the CLAT group; the mean composite TCM syndrome score was 21.15 points (SD 1.89) in the TLF group and 20.71 points (SD 2.32) in the CLAT group.

**TABLE 2:** Baseline characteristics of the rUTI participants at enrollment.

Item	TLF + CLAT (*n* = 98)	CLAT (*n* = 97)
Demographic characteristics		
Age (years), mean ± SD	52.24 ± 13.92	53.01 ± 13.90
Female [n (%)]	68 (69.39)	66 (68.04)
Medical history (months), mean ± SD	43.85 ± 14.24	43.06 ± 12.76
Disease characteristics [Case (%)]		
Urine white blood cells	94 (95.91)	92 (94.85)
Urine protein	95 (96.94)	93 (95.88)
Uropathogenic culture	96 (97.96)	96 (98.97)
Urine frequency	94 (95.91)	90 (92.78)
Urinary urgency	95 (96.94)	92 (94.85)
Urinary pain	92 (93.88)	91 (93.81)
Renal tubular injury markers, mean ± SD		
Urinary NAG/Cr (U/mmol)	3.14 ± 1.20	3.02 ± 1.19
Urinary β2-MG (mg/L)	0.43 ± 0.12	0.40 ± 0.15
Renal fibrosis markers, mean ± SD		
Urinary TGF-β1 (pg/ml)	25.56 ± 8.60	26.44 ± 8.97
Urinary MCP-1 (ng/L)	11.47 ± 3.40	11.90 ± 3.43
TCM syndrome score, mean ± SD	21.15 ± 1.89	20.71 ± 2.32

### 3.2 Efficacy results

The primary and secondary outcomes are shown in Table 3. The clinical effective rate of the two groups was calculated at week 12 of treatment. The recurrent rate of the two groups was calculated at weeks 4 and 12 post treatment, separately. Urinary NAG/Cr, β2-MG, TGF-β1, MCP-1, and TCM syndrome of the patients in the two groups were measured at weeks 0 and 12 of the treatment period.

**TABLE 3:** Primary and secondary outcomes.

Item	TLF + CLAT (*n* = 98)	CLAT (*n* = 97)	*p*-Value
Clinical efficacy [n (%)]			
Complete recovery	53 (54.08)	40 (41.24)**	<0.001
Significant recovery	30 (30.61)	27 (27.84)	-
Symptomatic improvement	6 (6.12)	5 (5.15)	-
No recovery	9 (9.19)	25 (25.77)**	<0.001
Recurrence rate [n (%)]			
At week 4 post treatment	14 (14.29)	35 (36.08)**	<0.001
At week 12 post treatment	19 (19.39)	46 (47.42)**	<0.001
Renal tubular injury markers, mean ± SD			
Urinary NAG/Cr (U/mmol)	1.74 ± 1.01	3.18 ± 1.03**	<0.001
Urinary β2-MG (mg/L)	0.22 ± 0.11	0.41 ± 0.20**	<0.001
Renal fibrosis markers, mean ± SD			
Urinary TGF-β1 (pg/ml)	17.51 ± 8.38	21.11 ± 8.59**	0.003
Urinary MCP-1 (ng/L)	6.68 ± 3.41	8.92 ± 3.46**	<0.001
TCM syndrome score, mean ± SD	6.22 ± 4.09	11.44 ± 5.52**	<0.001

*p < 0.05, **p < 0.01 vs. the TLF + CLAT group.

Urinary NAG/Cr was above the normal range before the treatment both in the TLF + CLAT group and CLAT group, with no significant difference (*p* = 0.116). After 12 weeks of treatment, NAG/Cr decreased in the TLF + CLAT group but not in the CLAT group (mean (SD) 1.74 (1.01) U. mmolCr (−1) vs. 3.18 (1.03) U. mmolCr (−1), respectively; *p* < 0.01). Similar changes were observed in urinary β2-MG (mean (SD) 0.22 (0.11) U. mmolCr (−1) vs. 0.41 (0.20) U. mmolCr (−1), respectively; *p* < 0.01).

**FIGURE 2:**
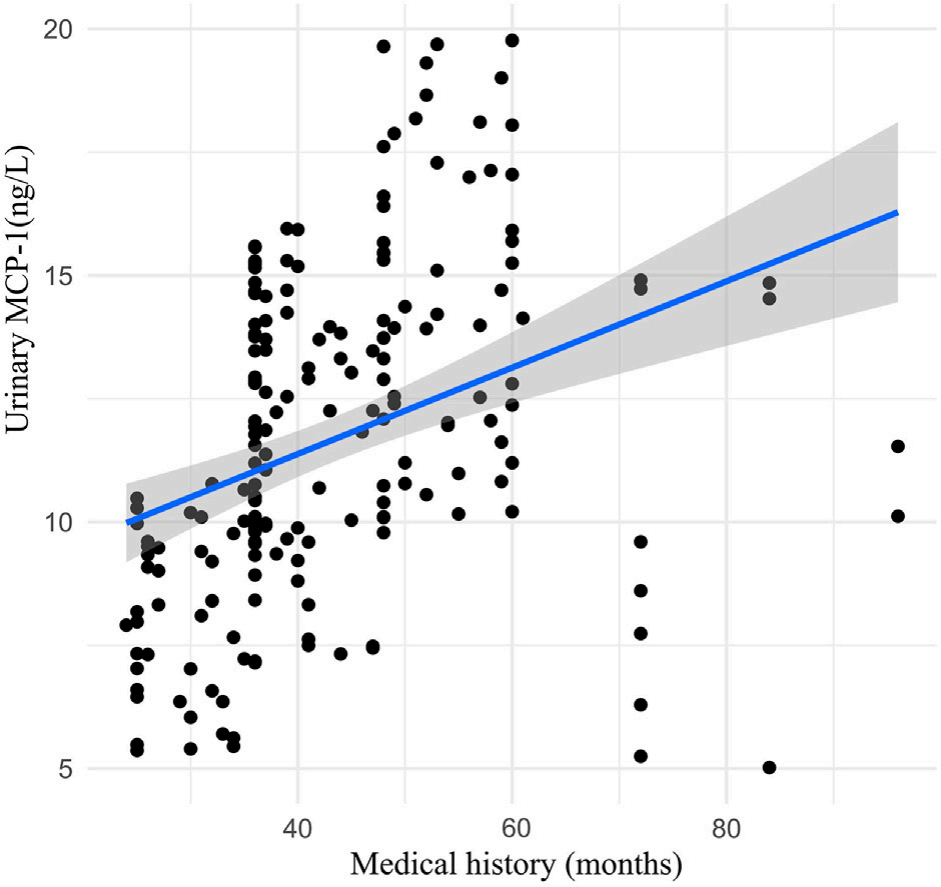
Correlation analysis between medical history (months) of rUTI patients and urinary MCP-1 (week 0).

**FIGURE 3:**
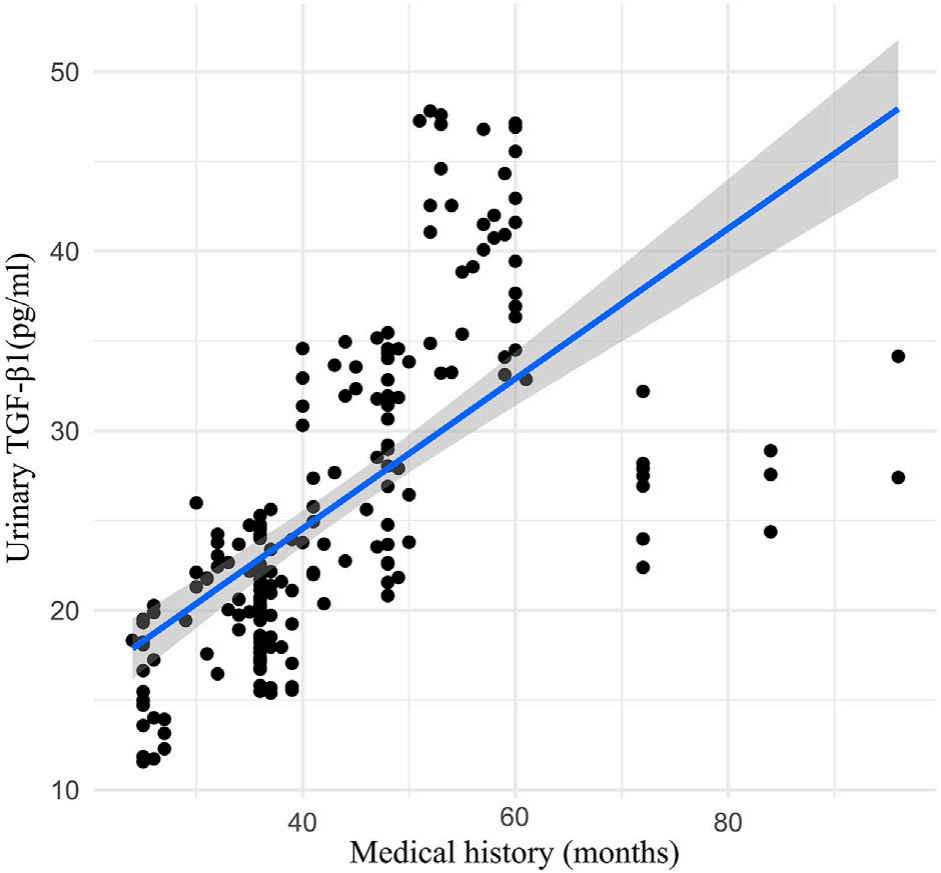
Correlation analysis between medical history (months) of rUTI patients and urinary TGF-β1 (week 0).

Urinary TGF-β1 decreased in the TLF + CLAT group but not in the CLAT group (mean (SD) 17.51 (8.38) pgml (−1) vs. 21.11 (8.59) pgml (−1), respectively; *p* < 0.05). Similar changes were observed in urinary MCP-1 (mean (SD) 6.68 (3.41) ngL (−1) vs. 8.92 (3.46) ngL (−1), respectively; *p* < 0.05). Additionally, a positive correlation within a certain range was shown in correlation analysis of medical history (months) of rUTI patients with urinary MCP-1 (r = 0.50, *p* < 0.05) and TGF-β1 (r = 0.78, *p* < 0.01) (Figures 2, 3).

### 3.3 Comprehensive efficacy

Compared with patients treated with CLAT alone, TLF combined with CLAT resulted in a higher clinical effective rate and lower recurrence rate and was sustained thereafter. For long-term clinical efficacy, the total effective rates were 90.82% (89/98) in the TLF + CLAT group and 74.23% (72/97) in the CLAT group, *p* < 0.05. For long-term clinical efficacy, the recurrent rate at week 4 post treatment was 14.29% (14/98) in the TLF + CLAT group and 36.08% (36/97) in the CLAT group; the recurrent rate at week 12 post treatment was 19.39% (19/98) in the TLF + CLAT group and 47.42% (46/97) in the CLAT group. Clinical efficacy is evaluated by the disappearance or improvement of clinical symptoms and uropathogenic clearance. More details could be found in Table 4.

**TABLE 4:** Evaluation of clinical efficacy.

Clinical efficacy	Indicators		
	Clinical symptom and sign	Urine routine (for two consecutive times)	Clean-catch midstream urine culture
Complete recovery	Disappeared	Normal	Negative
Significant recovery	Disappeared or nearly disappeared	Normal or almost normal	Negative
Symptomatic improvement	Alleviated	Significantly improved	Occasionally positive
No recovery	No significant improvement	No significant improvement	Colony count≥10^5^/ml 4 weeks after medication

Groups were similar in TCM syndrome scores before the treatment, with a mean composite symptom score of 21.15 points (SD 1.89) in the TLF + CLAT group and 20.71 points (SD 2.32) in the CLAT group. After 12 weeks of treatment, groups showed significant differences, with a mean composite symptom score of 6.22 points (SD 4.09) in the TLF + CLAT group and 11.44 points (SD 5.52) in the CLAT group, *p* < 0.01.

### 3.4 Safety results

There were no significant changes in the safety results, including blood routine, liver and kidney function, and electrocardiogram before and after treatment in the two groups, and no obvious adverse reactions occurred. CLAT-related laboratory abnormalities were not observed during this trial.

## 4 Discussion

In this study, we found that compared with CLAT, TLF combined with CLAT resulted in a significantly higher clinical effective rate and lower recurrence rate. Notably, additional benefits in protecting renal tubule, inhibiting renal fibrosis, and alleviating the non-infection-related physical signs and subjective symptoms were also observed in patients treated with TLF + CLAT. There were no obvious adverse reactions that occurred during this study.

CHM has been shown to be either used alone or combined with antibiotics, which may be more effective than antibiotics alone in treating (Yu et al., 2018). Equally, our previous studies showed relatively good clinical efficacy of TLF compared with CLAT in the treatment of rUTI (Gong et al., 2003; Gong et al., 2004; Gong et al., 2006; Gong et al., 2007; Gong et al., 2010). In the present study, we combined TLF with CLAT to treat rUTI and evaluated the clinical efficacy of this novel therapy. Compared with patients administrated with CLAT alone, a higher clinical effective rate was observed in TLF + CLAT group at week 12 of treatment. Furthermore, recurrent rates were monitored respectively at week 4 and week 12 post treatment to observe the long-term efficacy of the two treatments. Our results showed that TLF combined with CLAT has a longer and more stable effect; indirectly, it also suggested that TLF might have benefits to enhance the long-term efficacy.

RUTI involves inflammation of the urinary tract, which might result in renal tubular damage and ultimately kidney failure in those patients (Ichino et al., 2009; Lorenzo-Gomez et al., 2020). Urinary NAG and β2-MG are sensitive markers of tubular injury (Ix and Shlipak, 2021). The study has shown that abnormal expression of the urinary kidney injury marker (NAG) was also observed in rUTI patients (Lorenzo-Gomez et al., 2020). Therefore, quantitative detection of urinary renal tubular injury markers in patients with rUTI should be paid more attention, which might be helpful for early prevention of renal damage attributable to rUTI. In the present study, increased urinary renal tubular injury markers (NAG/Cr and β2-MG) were observed in rUTI patients and were reduced more after TLF + CLAT treatment compared with CLAT. Accordingly, TLF + CLAT might have an effect on renal tubular protection in rUTI patients.

Persistent inflammation associated with rUTI is a crucial inducement of IDRF, which is a complicated process involving proinflammatory and profibrotic paracrine mediators, such as growth factors, TGF-β1, MCP-1, and cytokines (Humphreys, 2018; Gu et al., 2020; Singh et al., 2021). As the critical profibrotic factors, urinary MCP-1 and TGF-β1 were monitored to evaluate the anti-fibrosis effects of the two treatments in the present study. Urinary TGF-β1 and MCP-1 were tested at week 0 and week 12 of treatment. Correlation analysis showed that the medical history (months) of rUTI patients was positively correlated with the level of profibrotic factors at week 0, suggesting that rUTI might be a risk factor for IDRF. Also, after 12 weeks of treatment, compared with CLAT, the expression of urinary TGF-β1 and MCP-1 decreased significantly in the TLF + CLAT group, which showed that TLF combined with CLAT has benefits in protecting against renal fibrosis. Therefore, attention should be paid to early prevention of renal fibrosis in the treatment of patients with rUTI. However, the specificity and sensitivity of urinary TGF-β1 and MCP-1 in the diagnosis of renal fibrosis attributable to rUTI still need more support from evidence-based clinical research, which already became a trigger for our subsequent experiments.

Furthermore, benefits in alleviating the non-infection-related physical signs and subjective symptoms were also observed in rUTI patients treated with TLF + CLAT. In clinical practice, we find that symptoms such as abdominal distention and fatigue would persist for a considerable time even after the infection is under control, which would result in significant inconvenience to those patients. The guidelines for rUTI showed that, since 2011, the non-infection-related physical signs and subjective symptoms have been recognized as part of the diagnosis criteria, along with “self-diagnosis of UTI” (Dason et al., 2011; Anger et al., 2019). This suggests that treatment of RUTI should not only focus on the negative result of urine culture but also pay attention to eradicate or alleviate the non-infection-related physical signs and subjective symptoms of rUTI patients. Therefore, this novel treatment is also a promising strategy helping to improve the health-related quality of life of rUTI patients.

In conjunction with the previous evidences (Gong et al., 2006; Gong et al., 2007; Gong et al., 2010; Lorenzo-Gomez et al., 2020), our results suggest that renal injury and IDRF might be the consequences due to rUTI in those patients, which involves complex interactions among multiple inflammatory cells and profibrotic cytokine signaling pathways. Given that, it is becoming more and more challenging to treat rUTI since it may not be possible to treat this disease by targeting one single molecular pathway. In other words, a multi-pharmacological approach involving several anti-inflammatory and antifibrotic molecules may need to be employed.

Chinese herbal is a natural medicine, contains a variety of effective ingredients, and has long been used to treat rUTI (Flower et al., 2015). TLF is an effective herbal prescription developed for treating rUTI. With the characteristics of the multi-ingredients, the mechanism of action and targets of Chinese herbal prescription TLF in patients with rUTI are also diverse. Studies by our group have shown the possible roles for TLF in the treatment of rUTI, including effects on reducing recurrence rate, alleviating symptoms, protecting renal tubular function, and inhibiting renal fibrosis (Gong et al., 2003; Gong et al., 2004; Gong et al., 2006; Gong et al., 2007; Gong et al., 2010). Consequently, TLF might be the multi-pharmacological approach with great promise in treating rUTI. However, further studies are still needed to investigate the mechanism of TLF against rUTI.

This study was designed as a double-blind RCT, with strict quality control processes for manufacturing TLF and placebo. The major contribution of our research is to demonstrate that rUTI is a significant threat that could cause renal damage and fibrosis, and TLF might be a multi-pharmacological approach involving anti-inflammatory and antifibrotic molecules and with great promise in treating rUTI. Since our study duration was relatively short (24 weeks), further investigation with longer follow-up is warranted to validate the role of urinary TGF-β1 and MCP-1 in the IDRF diagnosis and the impact of TLF in renal damage and fibrosis attributable to rUTI.

## 5 Conclusion

TLF combined with CLAT was superior to CLAT used alone in rUTIs in alleviating symptoms and decreasing recurrence and might have renal tubular protection and anti-fibrosis effects, which suggests TLF combined with CLAT might be an available treatment for rUTI. Moreover, TLF might be a multi-pharmacological approach involving anti-inflammatory and antifibrotic molecules and with great promise in treating rUTI.

## Data Availability

The original contributions presented in the study are included in the article/Supplementary Material; further inquiries can be directed to the corresponding author.
